# The effect of root canal treatment and post-crown restorations on stress distribution in teeth with periapical periodontitis: a finite element analysis

**DOI:** 10.1186/s12903-023-03612-9

**Published:** 2023-12-06

**Authors:** ShuoMin Chen, XinHua Hong, ZhangYan Ye, MengHan Wu, Liang Chen, LinMei Wu, Yilin Wang, YuGe Chen, JiaYu Wu, Jun Wang, QinHui Zhang, YuTian Wu, XiaoYu Sun, Xi Ding, ShengBin Huang, ShuFan Zhao

**Affiliations:** 1 Institute of Stomatology, School and Hospital of Stomatology, Wenzhou Medical University, No. 373, West Xueyuan Road, Lucheng District, Wenzhou, PR China; 2 Department of Prosthodontics, School and Hospital of Stomatology, Wenzhou Medical University, Wenzhou, China; 3 Department of Stomatology, Pingyang Hospital Affiliated of Wenzhou Medical University, Wenzhou, China; 4 Department of Dentistry, University of Alberta, Edmonton, Canada; 5 School of Medicine, Jiujiang University, Jiujiang, China; 6 Department of Periodontics, School and Hospital of Stomatology, Wenzhou Medical University, Wenzhou, China; 7 Department of Stomatology, the First Affiliated Hospital of Wenzhou Medical University, Ouhai District, Wenzhou, PR China; 8 Department of Oral Maxillofacial Surgery, School and Hospital of Stomatology, Wenzhou Medical University, No. 373, West Xueyuan Road, Lucheng District, Wenzhou, PR China

**Keywords:** Biomechanics, Finite element analysis, Periapical periodontitis, Post-crown restorations, Root canal treatment

## Abstract

**Aim:**

To evaluate the effects of root canal treatment (RCT) and post-crown restoration on stress distribution in teeth with periapical bone defects using finite element analysis.

**Methodology:**

Finite element models of mandibular second premolars and those with periapical bone defects (spherical defects with diameters of 5, 10, 15, and 20 mm) were created using digital model design software. The corresponding RCT and post-crown restoration models were constructed based on the different sizes of periapical bone defect models. The von Mises stress and tooth displacement distributions were comprehensively analyzed in each model.

**Results:**

**Overall analysis of the models**: RCT significantly increased the maximum von Mises stresses in teeth with periapical bone defects, while post-crown restoration greatly reduced the maximum von Mises stresses. RCT and post-crown restoration slightly reduced tooth displacement in the affected tooth. **Internal analysis of tooth**: RCT dramatically increased the maximum von Mises stress in all regions of the tooth, with the most pronounced increase in the coronal surface region. The post-crown restoration balances the internal stresses of the tooth and is most effective in periapical bone defect − 20-mm model. RCT and post-crown restoration slightly reduced the tooth displacement in all regions of the affected tooth.

**Conclusions:**

Root canal treatment seemed not to improve the biomechanical state of teeth with periapical bone defects. In contrast, post-crown restoration might effectively balance the stress concentrations caused by periapical bone defects, particularly extensive ones.

**Supplementary Information:**

The online version contains supplementary material available at 10.1186/s12903-023-03612-9.

## Introduction

Apical periodontitis (AP) is a common inflammatory condition that affects tissues surrounding the tooth root. It is typically caused by bacteria that enter the pulp of a tooth through cavities, cracks, or other types of damage, and then spread to the tissues surrounding the root [1–3]. Because the surrounding alveolar bone is destroyed, AP directly affects the stress condition of the affected tooth. This has a detrimental effect on the tooth’s ability to be preserved and restored. [4]. In a preliminary study, we found that the presence of periapical bone defects significantly affected the biomechanical response of teeth, the effects of which became more pronounced as the size of the bone defects increased. However, the subsequent therapeutic and restorative processes for teeth with periapical bone defects have not yet been studied in depth.

Conventional treatments for AP include root canal treatment (RCT), apical surgery, and medication. Although these treatments can remove the infection, they lead to a certain degree of stress concentration in the tooth, which may be detrimental to tooth preservation. [4–6]. In recent years, as nonsurgical approaches have been advocated for the treatment of AP [7, 8], preservation and restoration of the affected tooth at a later stage have become particularly important. Post-crown restoration is a common method of dental restoration that has been shown to be beneficial in improving the stress state of teeth [9] and is widely used in clinical settings. However, owing to imperfect treatment, teeth with periapical bone defects are frequently observed even after post-crown restoration [10, 11]. However, the effect of periapical bone defects caused by AP and their change in size on the subsequent post-crown restoration of the affected teeth has not been completely elucidated.

Finite element analysis (FEA) is a powerful tool in dental biomechanics that allows researchers and clinicians to better understand the mechanical behavior of dental structures [12]. Therefore, this study aimed to investigate the effect of RCT and post-crown restorations on the biomechanical state of teeth with periapical bone defects of different sizes by establishing corresponding FEA models and evaluate the mechanical stability of teeth with periapical bone defects after treatment and restoration. The null hypotheses for this study were as follows: (1) RCT and post-crown restoration do not change the biomechanical state of the affected tooth and (2) RCT and post-crown restoration result in the same biomechanical alterations in affected teeth with different sizes of periapical bone defects.

## Materials and methods

**Fig. 1:**
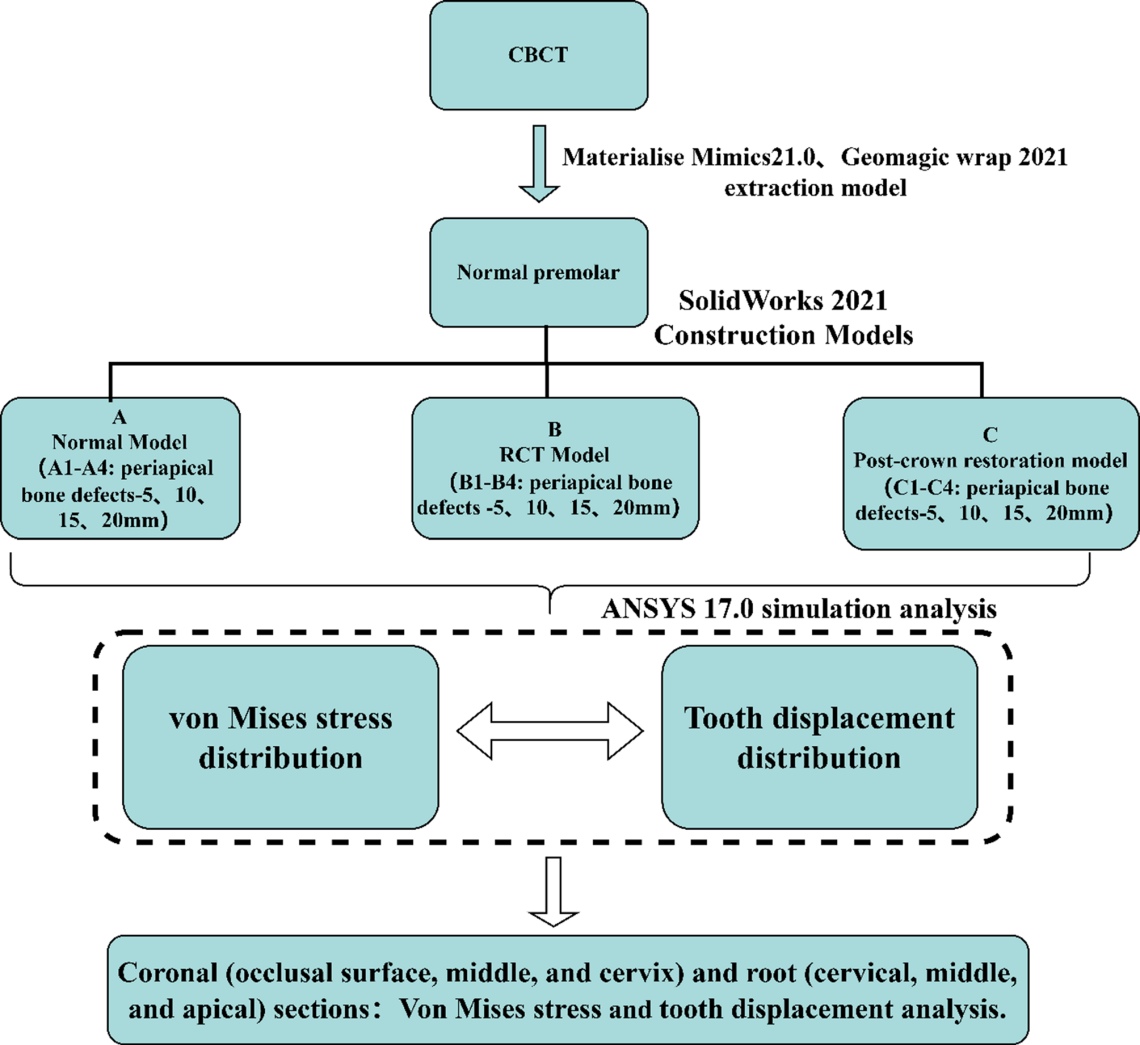
Schematic overview of the study design

The schematic illustration of the procedures performed sequentially is presented in Fig. 1.

### Cone-beam computed tomography (CBCT) data

**Fig. 2:**
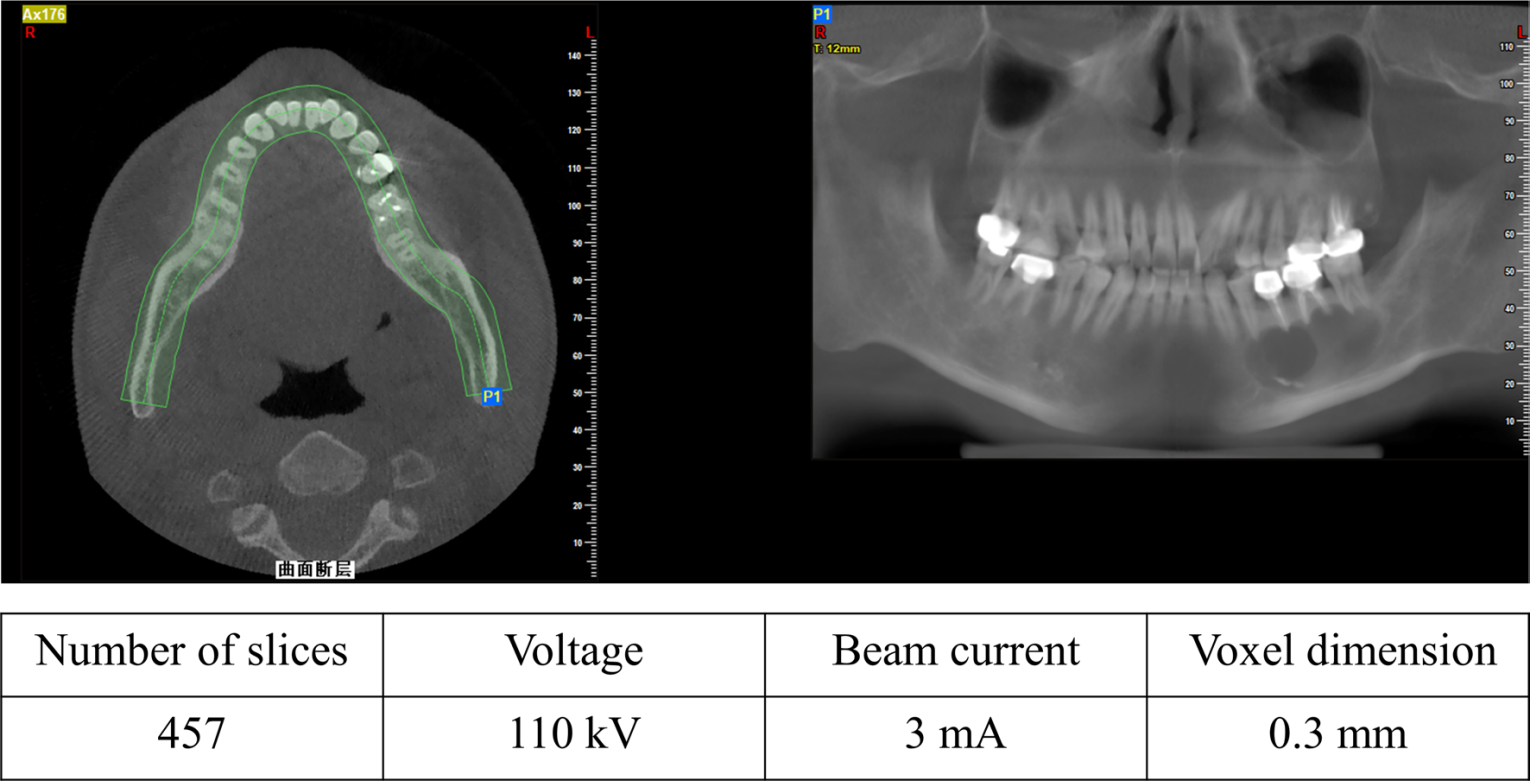
Typical CBCT images of periapical cysts after restoration

After obtaining informed consent from the volunteers, medical CBCT (iCRco, Inc., USA) digital image data of the oral and maxillofacial regions were obtained (Fig. 2). This study was approved by the Ethics Committee of Ethics Committee of the School and Hospital of Stomatology, Wenzhou Medical University Institute of Stomatology (Approval Number: WYYKQ2022022).

### Model construction

**Fig. 3:**
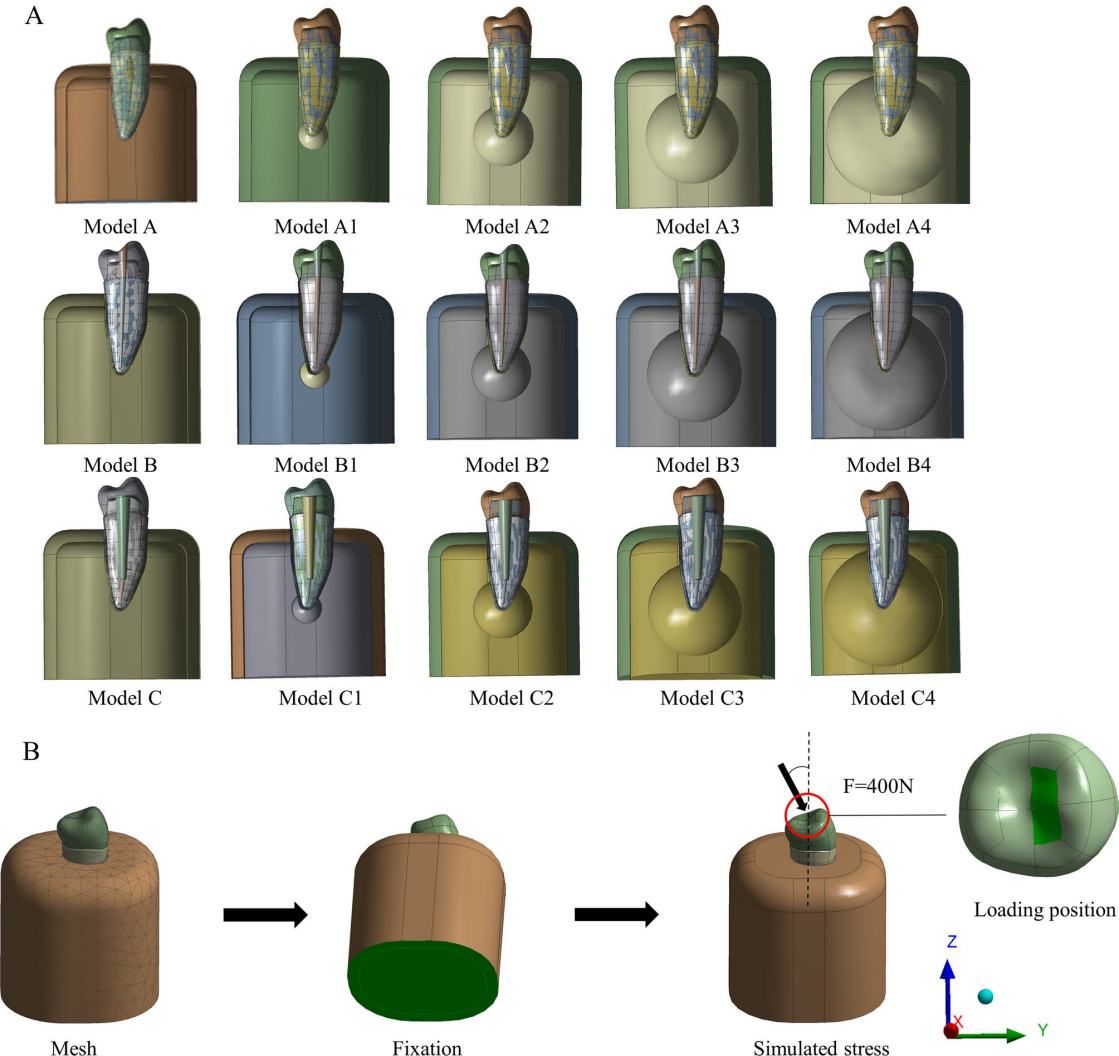
Schematic view of model structure and stress loading. **A**: Model A-A4 (periapical bone defect group). Models B-B4 (RCT group). Model C-C4 (post-crown restoration group). **B**: F = 400 N applied in a direction that was oblique (45°) to the model’s long axis

First, we successfully constructed a normal mandibular second premolar FEA model, including the cortical bone, cancellous bone, enamel, dentin, periodontal ligament, pulp, and cementum using Materialise Mimics 21.0 (Materialise, Belgium) and Geomagic Wrap 2021 (Geomagic, USA). We used SOLIDWORKS 2021 (Dassault Systèmes, France) to construct the following models (Fig. 3A):

(1) Model Group A

To simulate periapical bone defects caused by AP, a Model A periapical bone defects was constructed based on normal teeth (Model A). Model A1: Spherical defects with diameters of 5 mm surrounding the apical region. Model A2: Spherical defects with diameters of 10 mm surrounding the apical region. Model A3: Spherical defects with a diameter of 15 mm surrounding apical region. Model A4: Spherical defects with diameters of 20 mm surrounding the apical region.

(2) Model Group B

Construction of the corresponding RCT models (Model B) was based on the periapical bone defect model. The round root canal enlargement (MASTAL FILE#35, 0.04 taper), intra-root canal gutta-percha (Gutta Percha Points, GC Co., Japan) obturation, and tooth crown were sealed with 3 M resin (3 M, USA). The RCT Models B1, B2, B3, and B4 were further constructed based on the different sizes of the corresponding periapical bone defects.

(3) Model Group C

A post-crown restoration model (Model C) was constructed based on the RCT model. The post-tract preparation preserves the apical 4 mm of the gutta-percha and the later fiberglass post (3 M, USA) restoration, with a simulated 0.1-mm thickness bonding layer (3 M, USA) between the dentin and the fiberglass post. Starting from the anatomical contour of the crown (Wieland, Germany), the standard preparation process was followed, with a 2-mm preparation of the occlusal, buccal, and lingual surfaces, 1- to 1.5-mm preparation of the mesiodistal surfaces, 4–6° degree of polymerization, and 1-mm width of the shoulder. The post-crown restoration Models B1, B2, B3, and B4 were constructed based on the different sizes of the corresponding periapical bone defects.

All the models were meshed using second-order cells. The number of elements and nodes in each model are listed in Table 1.

**Table 1:** Number of nodes and elements for each model

Model	Mesh	1	2	3	4	Total
Model A	Nodes	84,274	83,921	83,504	82,980	334,679
	Elements	50,509	50,229	49,864	49,370	199,972
Model B	Nodes	83,699	83,449	82,977	82,390	332,515
	Elements	49,697	49,494	49,086	48,547	196,824
Model C	Nodes	93,031	92,947	92,600	91,932	370,510
	Elements	52,981	52,880	52,563	51,964	210,388

### Loading mode and loading force

In this study, the lingual surface of the crown received constant loading (400 N) that was applied in a direction that was oblique (45°) to the model’s long axis [13] (Fig. 3B). A simulation was performed to determine the stress state of the tooth during occlusion. The edges of the mandible were fixed to prevent movement in the X-, Y-, and Z-directions.

### Preconditions of the experiment

All tissues and materials were considered to have ideal bonding conditions and were isotropic, homogeneous, and linearly elastic. The corresponding mechanical parameters are listed in Table 2.

**Table 2:** Mechanical properties of the dental structures and restorative materials

Material	Young’s modulus (GPa)		Poisson’s ratio	Ref.
Cortical bone	13.7	0.3		[14]
Trabecular bone	1.37	0.3		[14]
Dentin	18.6	0.32		[14]
Cementum	8.2	0.3		[15]
Pulp	0.00207	0.45		[16]
Enamel	84.1	0.33		[16]
Periodontal ligament	0.05	0.49		[17]
Gutta-percha	0.00069	0.45		[4]
Composite resin	12	0.33		[4]
Resin cement	5.1	0.27		[18]
Crown	210	0.22		[19]
Fiberglass post	49	0.28		[19]

### Biomechanical analysis

ANSYS 17.0 (ANSYS, USA) was used to calculate the stress distribution and displacement pattern of the tooth. **Overall analysis of the models**: The overall stress distribution and tooth displacement for each model were obtained and their maximum von Mises stress and maximum tooth displacement calculated. **Internal analysis of the tooth**: The tooth was divided into coronal (occlusal surface, middle, and cervix) and root (cervix, middle, and apical) sections, and the stress distribution and tooth displacement in each section were analyzed.

## Results

### **Overall analysis of the model**

**Fig. 4:**
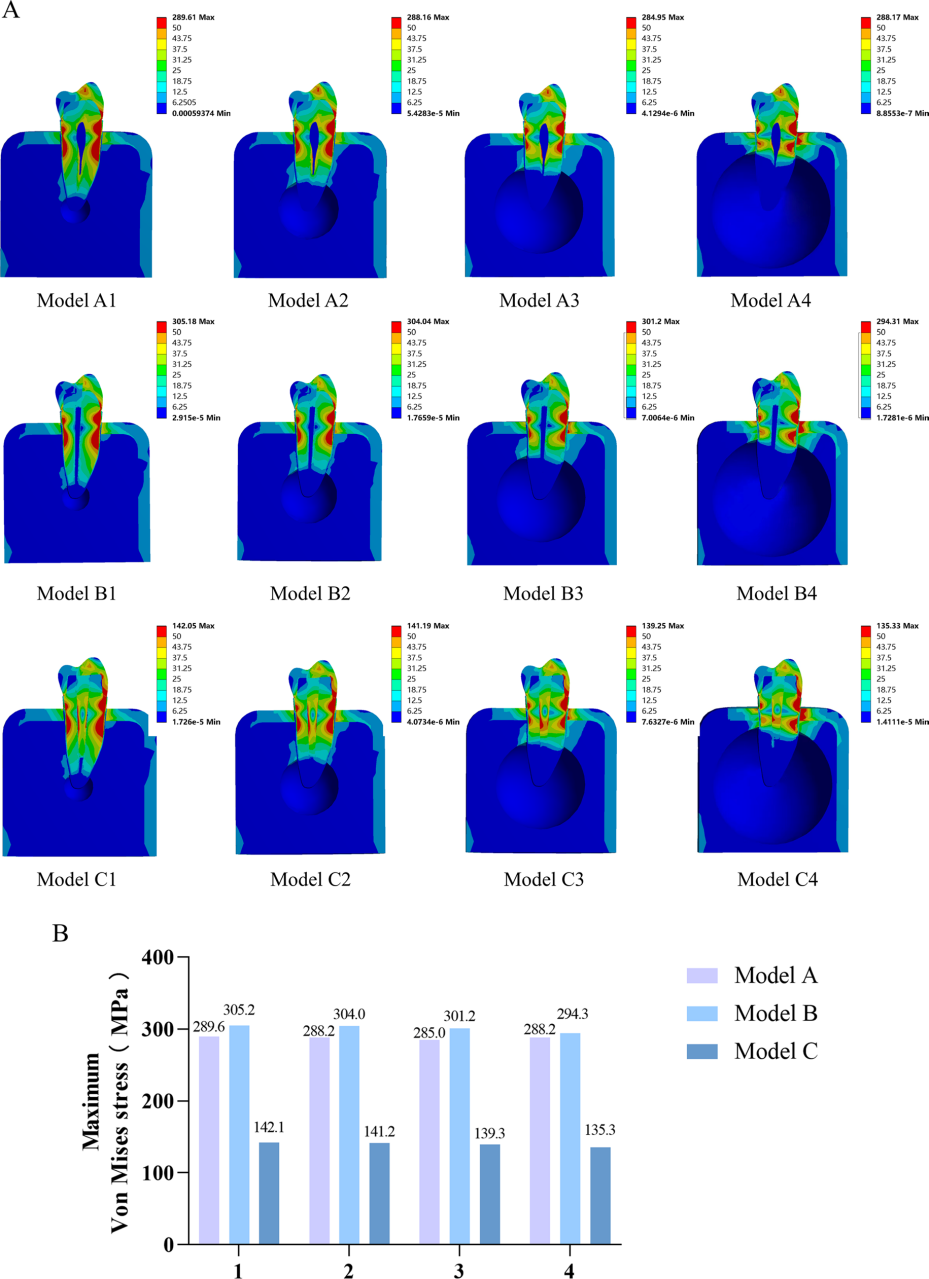
Stress distribution in overall analysis of the models. **A**: von Mises stress distribution cloud map of the model cross-section; **B**: model maximum von Mises stress analysis

Our FEA results were represented visually as the stress distribution and tooth displacement using an ANSYS 17.0 predefined progressive visual color scale (ANSYS, USA). Figure 4 shows the von Mises stress distribution cloud maps for the periapical bone defect models and corresponding RCT and post-crown restoration models.

Compared with the periapical bone defect model, RCT increased the maximum von Mises stresses of the teeth, whereas post-crown restoration greatly reduced the maximum von Mises stresses of the teeth (Fig. 4A, B). However, the differences between the RCT, post-crown restoration, and periapical bone-defect groups did not change as the size of the periapical bone defect increased (Fig. 4B). Statistical description of the stress distributions for each model in Supplementary Table 1.

**Fig. 5:**
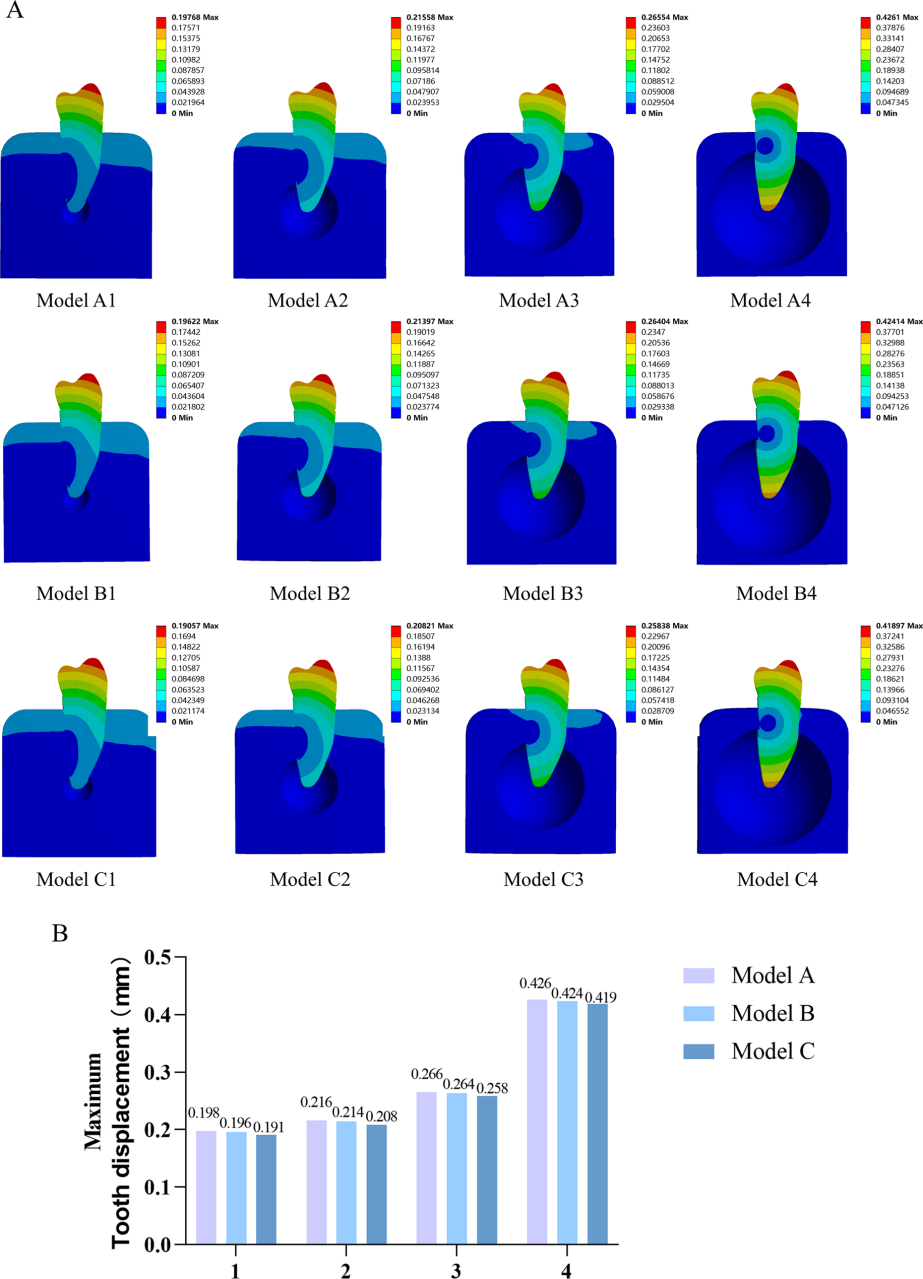
Tooth displacement distribution in overall analysis of the models. **A**: Tooth displacement distribution cloud map of the model cross-section; **B**: model maximum tooth displacement analysis

The tooth displacement of the root increased noticeably with an increase in the periapical bone defect size, particularly in the apical region, based on the tooth displacement distribution cloud maps of the periapical bone defect models (Fig. 5A Model A1-A4). Unlike the stress distribution, RCT and post-crown restoration only slightly reduced tooth displacement, and this effect did not change as the size of the bone defect increased (Fig. 5A, B). Statistical description of the tooth displacement for each model in Supplementary Table 2.

### Internal analysis of the tooth

**Fig. 6:**
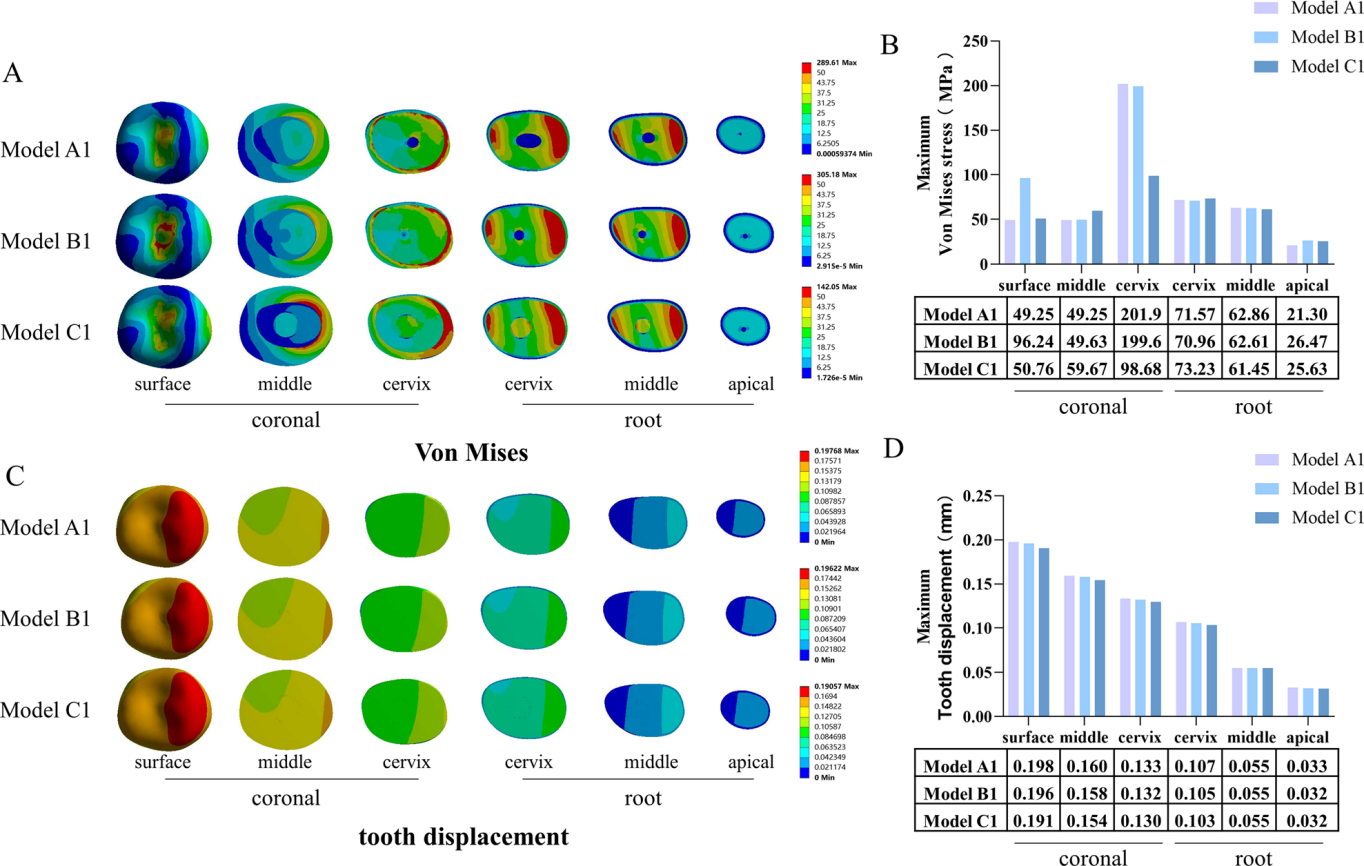
Von Mises stress and tooth displacement distribution in internal parts of the tooth—diameter of periapical bone defect: 5 mm. **A**: von Mises stress distribution cloud maps for internal parts of the tooth; **B**: maximum von Mises stress for internal parts of the tooth; **C**: tooth displacement distribution cloud maps for internal parts of the tooth; **D**: maximum tooth displacement for internal parts of the tooth

The von Mises stress distribution cloud maps and associated maximum von Mises stress analysis for the coronal (occlusal surface, middle, and cervix) and root (cervical, middle, and apical) sections revealed that in the coronal surface region, the maximum von Mises stresses in the RCT group greatly exceeded those in the periapical bone defect and post-crown restoration groups (Fig. 6A, B). In the coronal cervix, the maximum von Mises stress in the post-crown restoration group decreased considerably, whereas the maximum von Mises stress in the rest of the region increased slightly. In addition, similar results were obtained in the internal analysis of all models with different sizes of periapical bone defects (Supplementary Figs. 1, 2, 3).

The cloud maps of the tooth displacement distribution and the corresponding maximum tooth displacement analysis revealed that, in all regions of the tooth interior, the RCT group showed a decrease in tooth displacement in all areas of the tooth interior, which was more pronounced in the pile core crown restoration group (Fig. 6C, D). Internal analysis of all models showed similar results for periapical bone defect diameters of 5, 10, and 15 mm. However, when the periapical bone defect diameter was 20 mm, the tooth displacement in the apical region of the post-crown restoration group significantly decreased (Supplementary Fig. 3).

## Discussion

According to previous studies, AP causes periapical bone resorption, which reduces the biomechanical resistance of the affected tooth and makes it more susceptible to vertical root fracture [20–22]. RCT and post-crown restorations are widely used in clinical treatment and restoration. In this study, we simulated the treatment and restoration of teeth with periapical defects by combining clinical situations and procedures, such as RCT and post-crown restorations, using FEA. Our results showed that (1) RCT led to increased stress concentration, and post-crown restoration greatly reduced stress, and (2) this effect changed as the size of the bone defect increased. Therefore, the null hypothesis was rejected.

### FEA models reasonableness analysis

RCT, as the primary treatment modality for teeth with AP, has also been shown to be useful for the treatment of large AP [7, 8, 23]. The American Dental Association (ADA) recommends that root canals should be prepared to a depth that extends to the tip of the tooth root when performing RCT [24]. Concurrently, the root canal preparation of the premolar needs to be enlarged to a diameter of about 1.2–1.5 mm to ensure complete removal of pulp and necrotic tissue inside the root canal and provide sufficient space for subsequent filling [25]. Therefore, we constructed an access cavity based on the abovementioned criteria and removed the entire apex of the pulp chamber to create a straight path from the access port to the root canal orifice. We chose gutta-percha as the root canal filling material because of its good apical filling properties, which are widely used in clinical practice. Studies have shown that good apical filling can minimize apical condensation force during root canal therapy [26].

Post-crown restorations can improve the health, function, and appearance of teeth while providing a durable and long-lasting solution to dental issues [27, 28]. The post-tract preparation preserves the apical 4 mm of the gutta-percha, [29]and the morphology of the post-tract preparation should match the chosen post type and conform to physiological anatomical principles [30]. Recently, various forms of post restoration in clinical applications, such as fiberglass posts, one-piece custom-milled zirconia posts, and cast Ni–Cr posts exist [31]. This study used fiberglass posts for post restoration because of their high clinical usage rate. We chose zirconia crowns because studies have shown that they are good restorative material owing to their excellent aesthetic and physical properties [32]. The Young’s modulus and Poisson’s ratio of zirconia crowns are 210 GPa and 0.22, respectively, while Young’s modulus for metal-ceramic crowns is approximately 70–100 GPa, metal crowns have a Young’s modulus of approximately 100–200 GPa. [33]. It may indicate that zirconia crowns are more rigid and less flexible than metal-ceramic crowns and metal crowns. Meanwhile, previous study demonstrated that the tooth restored with zirconia crowns may be more resistant to fracture and wear, as they can better withstand the occlusal load without bending or cracking [34].

In this experiment, the structure, material selection, and parameter settings of the model construction are referred to in relevant finite element studies and reasonably adjusted according to the clinical situation; thus, the model construction is reasonable.

### Von Mises stress analysis

In the overall analysis, RCT increased the maximum von Mises stresses of the teeth, while post-crown restoration greatly reduced the maximum von Mises stresses of the teeth (Fig. 4A, B), Studies have demonstrated that the cavity inside the tooth becomes larger during RCT because the pulp and nerve are removed, which makes the tooth’s structure more fragile as well [5, 35]. Therefore, recently, the concept of minimally invasive endodontics (MIE) has been proposed as a means to enhance the fracture resistance of teeth that have undergone RCT [36]. For mature teeth, the gutta-perch filling root canal system is commonly used in RCT. However, the application of apical force to the gutta-percha causes pressure on the material and, as a result, circumferential tensile stresses on the canal surface. These stresses can cause vertical root fractures (VRF) [26, 37]. In contrast to mature teeth, mineral trioxide aggregate (MTA) may be a more suitable material for RCT in immature teeth because of the shorter treatment time associated with its use [38]. However, whether this change in the filling material of the root canal system affects the tooth root mechanics requires further study.

In contrast, post-crown restoration can significantly improve the mechanical properties of the affected tooth through the reinforcement of the post and the protective effect of the crown; thus, it is widely used in the preservation and restoration of affected teeth [39, 40]. Previous studies on post-crown restorations have focused on restorative materials and methods without considering periapical bone changes in teeth with AP [41]. However, periapical bone defects are often observed in teeth with AP in the clinic [10]. Consistent with the results of normal teeth with post-crown restorations, our study showed that post-crown restoration remains an effective restorative method even when periapical bone defects are present. According to data obtained from a retrospective study, the likelihood of root fractures occurring within 3 years of receiving zirconia post-crown restoration was 1.19% [42]. This is because of the application of post-improved mechanical stability of teeth. High-strength and high-hardness materials, such as zirconia, are used for restoration, which can significantly increase the load-bearing capacity of the teeth.

To understand the internal stress changes in periapical bone-defective teeth after RCT and post-crown restoration, we further investigated von Mises stress changes in six parts of the tooth in the coronal (occlusal surface, middle, and cervix) and root (cervix, middle, and apical) sections. We found that the RCT group showed stress concentration at the occlusal surface due to the partial loss of tissue in the coronal region of the tooth due to RCT. The composite resin was more brittle than the enamel and thus could not accommodate higher stresses internally [43, 44]. The stresses in the cervical region of the root decreased considerably after the post-crown restoration, although the von Mises stresses in other areas of the tooth increased slightly (Fig. 6A, B). This may be due to the disappearance of the internal cavity of the tooth after the post-crown restoration, which resulted in a more uniform stress distribution. This mechanical change is beneficial for tooth preservation [45, 46].

### Tooth displacement analysis

The maximum tooth displacement was slightly lower in the RCT and post-crown restoration groups than in the periapical bone defect group under different periapical bone defect conditions. This has a positive effect on tooth preservation. It has been demonstrated that excessive tooth displacement can cause damage to periodontal tissues or even fracture of the tooth itself [4]. This phenomenon may be related to the homogenization of the internal stress distribution after RCT and post-crown restoration of the tooth. In addition, in the internal analysis of the teeth, the tooth displacement in the apical region of the post-crown restoration group showed a precipitous decrease when the periapical bone defect was 20 mm (Supplementary Fig. 3), suggesting that post-crown restoration exerts a more pronounced effect on the preservation and restoration of teeth with extensive periapical bone defects.

However, in our study, RCT and post-crown treatment were ineffective in reducing tooth displacement in teeth with periapical bone defects. This may be due to the fact that periapical bone loss results in the loss of alveolar bone support and restriction of the tooth root [47]. The alveolar bone is the part of the jawbone that surrounds and supports the teeth. It plays a crucial role in maintaining healthy teeth by providing a rigid foundation for tooth roots to anchor into [48]. Therefore, for the restoration of teeth with periapical bone defects, other treatments, such as periapical bone-defect filling materials, are suggested to increase the support of the affected tooth [49]. However, differences in the restorative effects of different bone-filling materials exist, which requires further research.

### Limitations of the current study

This study found that RCT does not improve the biomechanical state of teeth with periapical bone defects, and post-crown restoration can effectively balance stress concentrations and provide suggestions for the prevention and treatment of teeth with periapical bone defects. Some limitations to the design of the experiment are as follows: 1) This experiment chose gutta-percha because of its good biocompatibility and plasticity. Recently, root canal filling materials have more options, such as calcium silicate-based cement and bioceramics [50]. Equally noteworthy is the fact that, a sufficient bond strength at the material-dentin contact is essential to guarantee a perfect seal of the root canal system and strengthen the resistance of the cervical section of the tooth to prevent fracture [51]. Meanwhile, in a clinical study, MTA plug and bond restoration was found to be a good dental restoration to prevent or minimizing microleakage [52]. Therefore, mechanical properties of different root canal filling materials and bond at the material-dentin interface needs to be more fully considered in the subsequent studies.

2) The experiment was well constructed for each structure of the teeth and alveolar bone and effectively simulated their intra-oral biomechanical conditions. However, this study did not simulate dental osseous structures as a hydrostatic system and a Functionally Graded Material Property system. In future studies, constructing different dental models and designing in vitro validation experiments to restore the intraoral conditions more accurately are necessary.

## Conclusion

Within the limitations of the present study and those of finite element analysis, it was possible to conclude that root canal treatments seemed not to reduce the stress concentration caused by periapical bone defects. In contrast, post-crown restorations might effectively balance the uneven stress distribution within teeth with periapical bone defects, and improves the stability of the tooth stability. Root canal treatment and post-crown restorations might reduce slightly the increased tooth displacement of the affected tooth due to periapical bone defects. However, these results should be further validated by in vivo studies.

### Electronic supplementary material

Below is the link to the electronic supplementary material.

Supplementary Material 1

Supplementary Material 2

## Data Availability

All data generated or analyzed during this study are included in this published article.
