# Supplementary material for: The effect of root canal treatment and post-crown restorations on stress distribution in teeth with periapical periodontitis: a finite element analysis
Source: BMC Oral Health. 2023 Dec 6;23:973. doi: 10.1186/s12903-023-03612-9 (PMC10701996; doi:10.1186/s12903-023-03612-9)
Supplement: Supplementary file 1 — Supplementary Material 1 [file 12903_2023_3612_MOESM1_ESM.docx]

**Supplementary table 1** **Statistical description of the stress distributions (Mpa) for each model**

| Model | Aver. | SD | Min. | 1st Quart. | Median | 3rd Quart. | Max. |
| --- | --- | --- | --- | --- | --- | --- | --- |
| Model A1 | 19.04 | 17.16 | 0 | 5.713 | 15.83 | 28.22 | 289.6 |
| Model A2 | 17.92 | 17.02 | 0 | 5.077 | 14.05 | 27.3 | 288.2 |
| Model A3 | 16.71 | 16.84 | 0 | 3.587 | 12.71 | 25.81 | 285 |
| Model A4 | 15.58 | 16.9 | 0 | 1.289 | 11.31 | 24.35 | 288.2 |
| Model B1 | 19.08 | 16.88 | 0 | 6.095 | 16.04 | 27.7 | 305.2 |
| Model B2 | 17.89 | 16.74 | 0 | 5.355 | 14.26 | 26.76 | 304 |
| Model B3 | 16.7 | 16.55 | 0 | 3.972 | 12.94 | 25.43 | 301.2 |
| Model B4 | 15.58 | 16.63 | 0 | 1.734 | 11.6 | 24.01 | 294.3 |
| Model C1 | 18.16 | 16.1 | 0 | 4.327 | 14.73 | 28.08 | 142.1 |
| Model C2 | 17.03 | 15.86 | 0 | 3.763 | 12.85 | 27.07 | 141.2 |
| Model C3 | 15.69 | 15.63 | 0 | 1.934 | 11.03 | 25.64 | 139.3 |
| Model C4 | 14.42 | 15.64 | 0 | 0.6305 | 9.144 | 23.81 | 135.3 |

Statistical description in detail the Von Mises stress of each of the models（including average, median, standard deviation, min, max, 1st Quart. and 3rd Quart.）.

**Supplementary table 2 Statistical description of the tooth displacement (mm) for each model**

| Model | Aver. | SD | Min. | 1st Quart. | Median | 3rd Quart. | Max. |
| --- | --- | --- | --- | --- | --- | --- | --- |
| Model A1 | 0.06604 | 0.05077 | 0 | 0.0246 | 0.0459 | 0.107 | 0.1972 |
| Model A2 | 0.07419 | 0.05386 | 0 | 0.0317 | 0.0548 | 0.1164 | 0.2156 |
| Model A3 | 0.097 | 0.06317 | 0 | 0.0476 | 0.0862 | 0.1402 | 0.2655 |
| Model A4 | 0.1719 | 0.1023 | 0 | 0.0901 | 0.1682 | 0.2502 | 0.4261 |
| Model B1 | 0.06573 | 0.05108 | 0 | 0.0242 | 0.0449 | 0.1076 | 0.1962 |
| Model B2 | 0.0739 | 0.05391 | 0 | 0.0311 | 0.0541 | 0.1167 | 0.214 |
| Model B3 | 0.09688 | 0.06345 | 0 | 0.0463 | 0.0861 | 0.1411 | 0.264 |
| Model B4 | 0.172 | 0.1032 | 0 | 0.088 | 0.1685 | 0.2527 | 0.4241 |
| Model C1 | 0.06316 | 0.04883 | 0 | 0.0244 | 0.042 | 0.103 | 0.1906 |
| Model C2 | 0.07189 | 0.0511 | 0 | 0.0332 | 0.0523 | 0.1122 | 0.2082 |
| Model C3 | 0.09631 | 0.05994 | 0 | 0.05 | 0.0873 | 0.1358 | 0.2584 |
| Model C4 | 0.1762 | 0.1006 | 0 | 0.0942 | 0.1756 | 0.2573 | 0.419 |

Statistical description in detail the tooth displacement of each of the models（including average, median, standard deviation, min, max, 1st Quart. and 3rd Quart.）.
