# Supplementary material for: The effect of root canal treatment and post-crown restorations on stress distribution in teeth with periapical periodontitis: a finite element analysis
Source: BMC Oral Health. 2023 Dec 6;23:973. doi: 10.1186/s12903-023-03612-9 (PMC10701996; doi:10.1186/s12903-023-03612-9)
Supplement: Supplementary file 2 — Supplementary Material 2 [file 12903_2023_3612_MOESM2_ESM.docx]

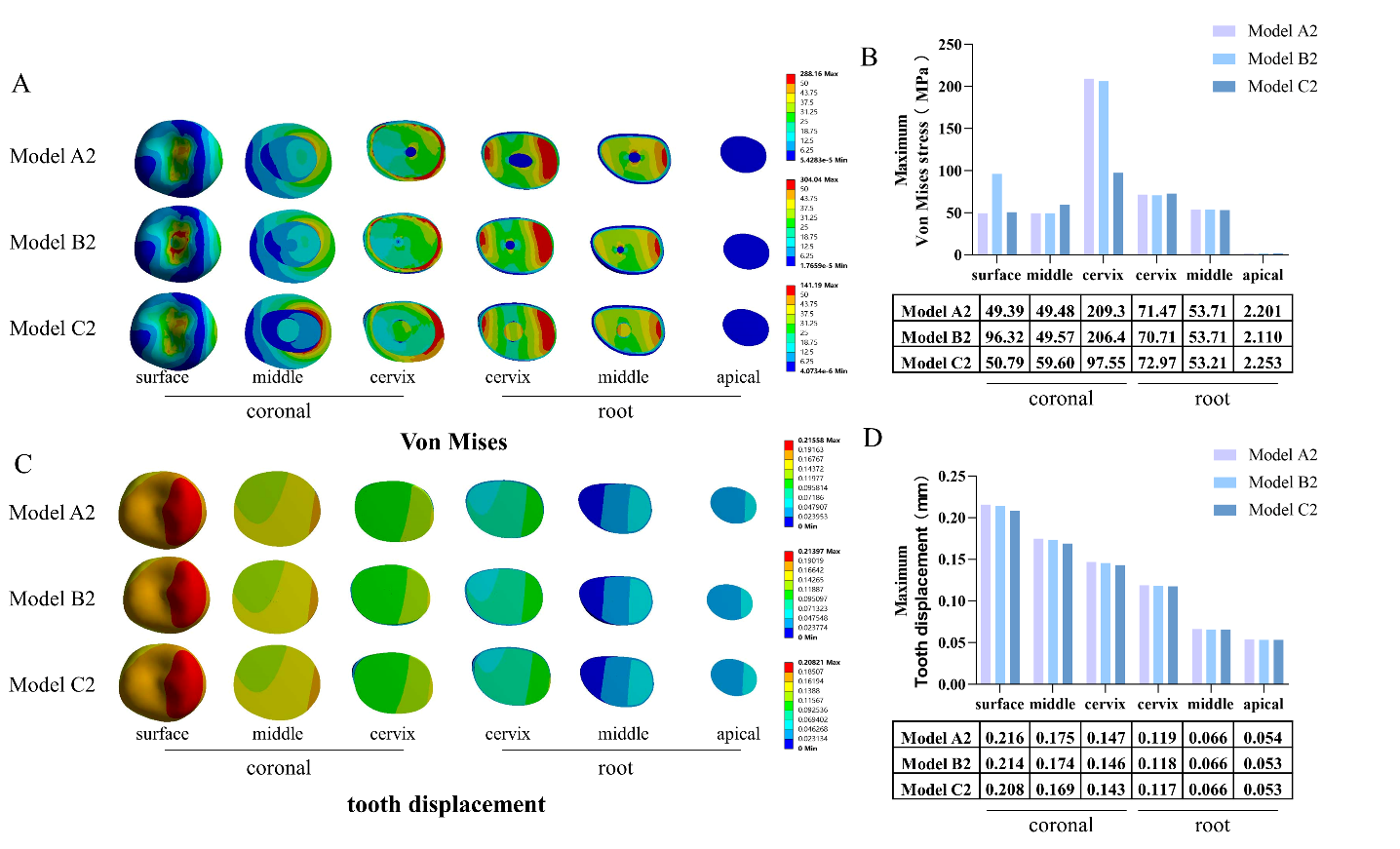


Supplementary Figure1. Von Mises stress and tooth displacement distribution in internal parts of the tooth—diameter of periapical bone defect: 10 mm.

A: von Mises stress distribution cloud maps for internal parts of the tooth; B: maximum von Mises stress for internal parts of the tooth; C: tooth displacement distribution cloud maps for internal parts of the tooth; D: maximum tooth displacement for internal parts of the tooth.


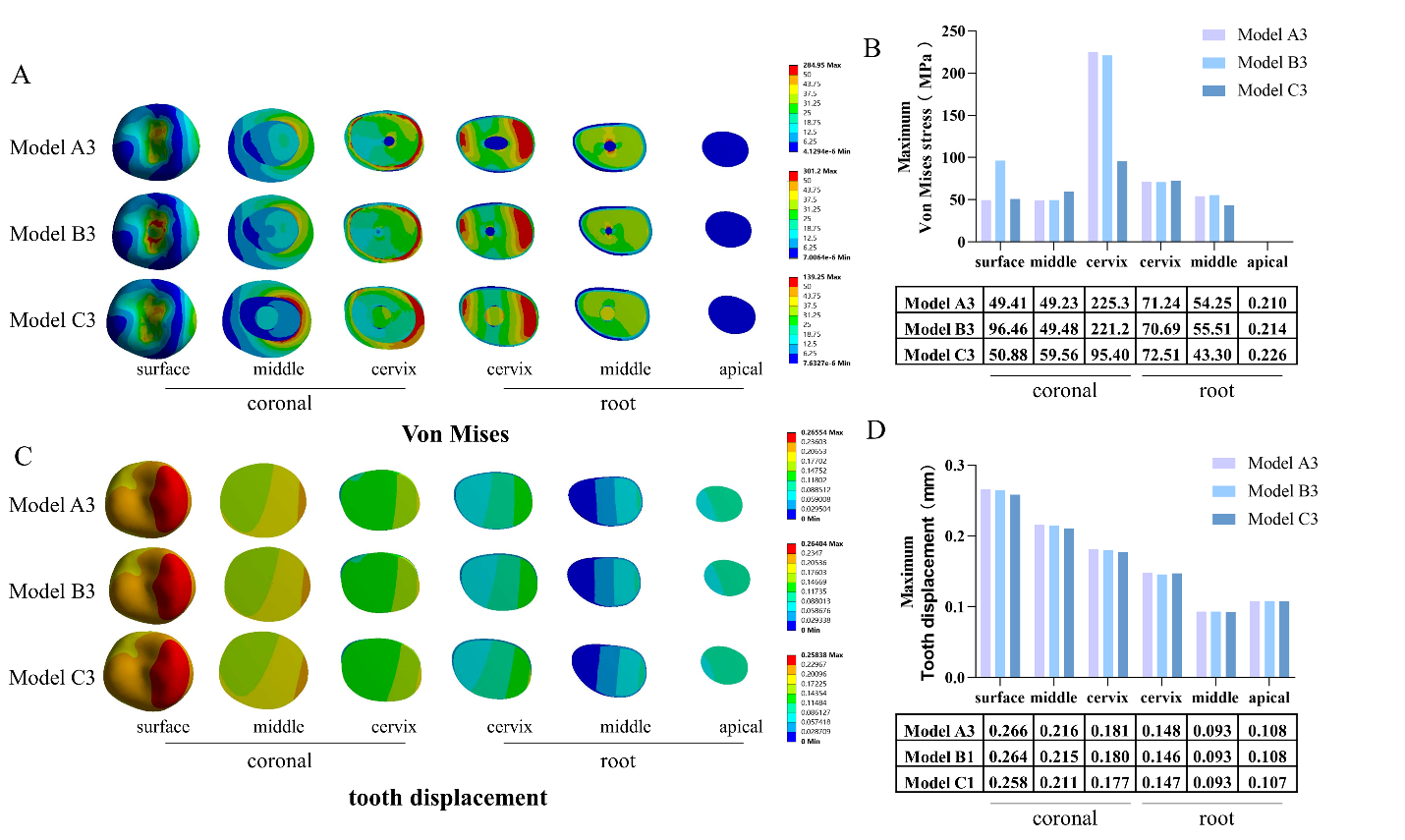


Supplementary Figure 2. Von Mises stress and tooth displacement distribution in internal parts of the tooth—diameter of periapical bone defect: 15 mm.

A: von Mises stress distribution cloud maps for internal parts of the tooth; B: maximum von Mises stress for internal parts of the tooth; C: tooth displacement distribution cloud maps for internal parts of the tooth; D: maximum tooth displacement for internal parts of the tooth.


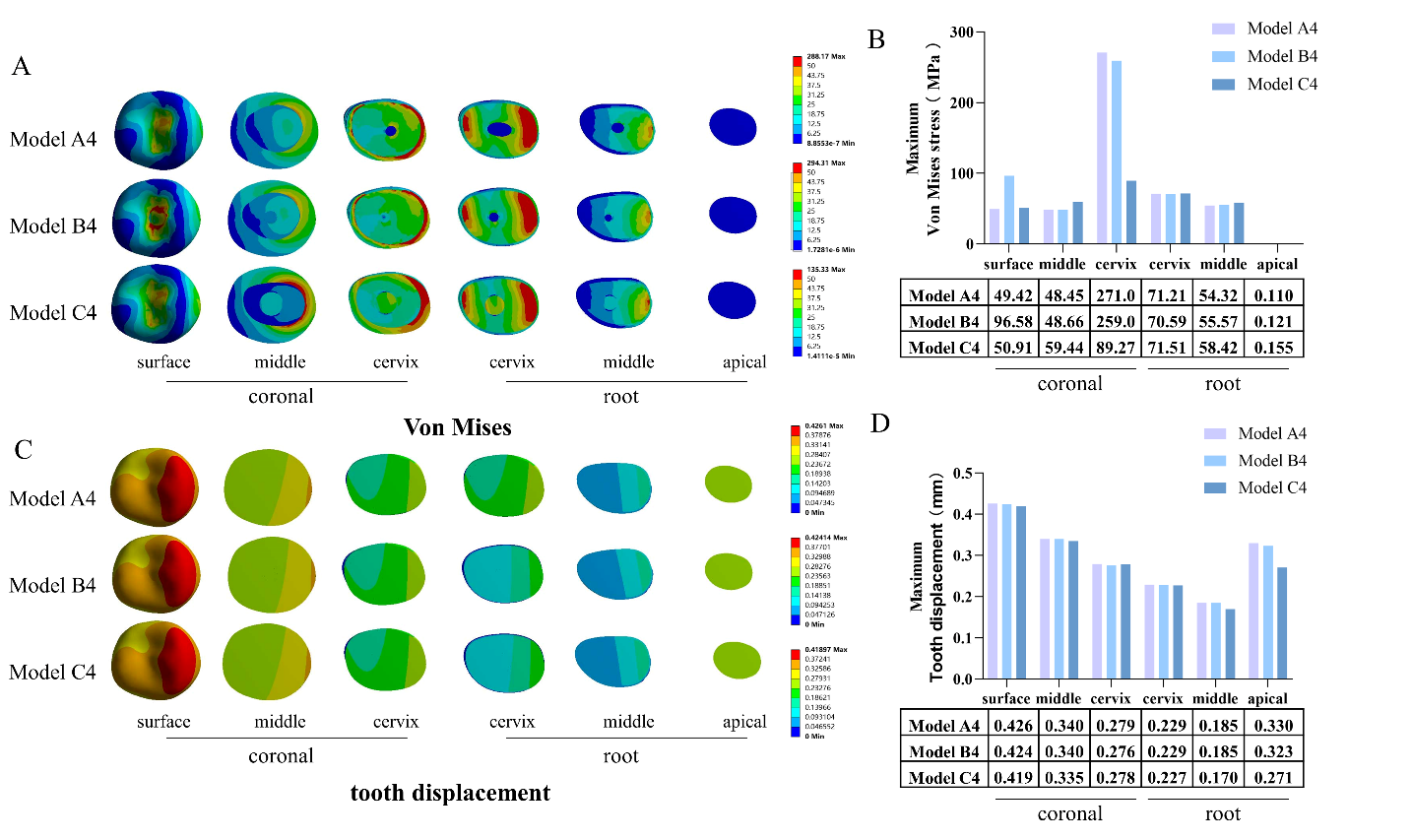


Supplementary Figure 3. Von Mises stress and tooth displacement distribution in internal parts of the tooth—diameter of periapical bone defect: 20 mm.

A: von Mises stress distribution cloud maps for internal parts of the tooth; B: maximum von Mises stress for internal parts of the tooth; C: tooth displacement distribution cloud maps for internal parts of the tooth; D: maximum tooth displacement for internal parts of the tooth.
